# Self-prescribing among young Norwegian doctors: a nine-year follow-up study of a nationwide sample

**DOI:** 10.1186/1741-7015-3-16

**Published:** 2005-10-21

**Authors:** Erlend Hem, Guro Stokke, Reidar Tyssen, Nina T Grønvold, Per Vaglum, Øivind Ekeberg

**Affiliations:** 1 Department of Behavioural Sciences in Medicine, Institute of Basic Medical Sciences, University of Oslo, PO Box 1111 Blindern, NO-0317 Oslo, Norway

## Abstract

**Background:**

Self-prescribing among doctors is common, but no longitudinal studies have documented this issue. We studied the self-prescribing behaviour among young Norwegian physicians and the predictors of self-prescribing.

**Methods:**

We conducted a nationwide, prospective and longitudinal study following young Norwegian physicians from internship through the subsequent nine years using three postal questionnaires. Chi-square tests and logistic regression models were applied.

**Results:**

About 54% of the physicians in their fourth and ninth postgraduate years had self-prescribed medication at least once during the previous year. Among those who had used prescription medication during the previous year, about 90% had self-prescribed. Self-prescribing behaviour did not differ significantly between men and women, or according to the type of work at any time. The most frequently self-prescribed medications were antibiotics (71%–81%), contraceptives (24%–25%), analgesics (18%–21%), and hypnotics (9%–12%). Those who had needed treatment for mental problems had self-prescribed hypnotics and sedatives to a greater extent than the others. Being male, having self-prescribed during internship, somatic complaints, mental distress, subjective health complaints, and not having sought help from a general practitioner, were significant adjusted predictors of self-prescribing in the ninth postgraduate year.

**Conclusion:**

The level of self-prescribing among young Norwegian physicians is relatively high, and this behaviour is established early in their professional lives. Although self-prescribing is acceptable in some situations, physicians should seek professional help for illness. Efforts to inculcate more rational help-seeking behaviour should probably start in medical schools.

## Background

Doctors commonly self-prescribe medication, and estimates of the prevalence of self-prescribing vary widely from 39% to 99% [1-6]. Self-prescribing may be a matter of concern for several reasons. First, there is a lack of objectivity and professional distance, which normally exist in a physician-patient relation, and self-treatment can lead to delayed diagnosis and treatment [7] and worsening of the illness [8]. Second, many diseases need follow-up apart from medication, particularly for mental illness and chronic diseases. Third, self-prescribing may be an indicator that the doctor is neglecting his or her own health. Ethical rules for doctors in several countries explicitly state the importance of seeking help when ill [9,10]. Finally, the potential for addiction is the main concern when self-prescribing is discussed [11]. This topic is important because the health habits and attitudes of physicians influence the counselling and care they provide to patients [12].

The obstacles to seeking professional care are many and complex, and include both rational and irrational factors [13]. Many physicians find it difficult to enter the patient role (role reversal) for various individual and organizational reasons [8,11], such as time pressure, the stigmatizing nature of sickness, worries about bothering or letting down colleagues, fear of showing weakness or lack of medical knowledge, concerns about confidentiality, and fear of restrictions of medical licensing [14-16].

Several papers have been published on self-treatment among doctors in various countries [1-8,17-26]. Some findings are consistent across countries, such as a high level of self-treatment and a reluctance to seek professional care. However, the literature is ambiguous on several aspects of doctors' self-care.

Older doctors have a high prevalence of self-treatment [19,25], but young physicians also frequently self-treat [3,8,20]. Some studies have shown no gender differences in self-treatment [3,8,25], whereas others have found that female physicians use health services more than their male colleagues [23,24]. The influence of a medical specialty is also inconsistent: some studies have shown that general practitioners are more likely to self-treat than hospital physicians [20,25], others have found only small differences [7,19], and still others have shown that clinicians working outside a hospital are significantly less likely to undertake self-treatment than hospital physicians [8]. A substantial part of self-prescribing involves treating minor illnesses and acute infections. However, in Finland, one of the most common reasons for physician self-medication is mental disorder or insomnia [24], and more than 25% of American physicians have self-prescribed a psychoactive drug [4,27].

An important reason for the differences in results among studies is that most have been limited to small, selected or non-representative samples, such as general practitioners or graduates from one medical school. To our knowledge, all previous studies have been cross-sectional. We conducted a prospective longitudinal study of a nationwide sample of medical students from all four universities in Norway and assessed their self-prescribing behaviour in a nine-year follow-up study.

Self-prescribing is common among residents [4,28]. The aim of this study was to investigate the development of such behaviour during internship and the first nine postgraduate years, and to identify the predictors for self-prescribing. We have previously shown that a substantial proportion of doctors report mental health problems requiring treatment [13]. We were interested in whether these physicians are more likely to self-prescribe psychotropic medication than others, and thereby constitute a risk group for future misuse of drugs and addiction.

We believe that various symptoms and diseases might be important to self-prescribing behaviour, and included three symptom and disease measures in the multivariate analysis, including subjective health complaints with a particular focus on musculoskeletal problems, eight somatic disease categories, and mental distress tapping anxiety and depressive symptoms. Finally, we presumed that those who had sought help from a general practitioner would self-prescribe to a lesser extent than others. We asked the following specific research questions:

• What is the prevalence of self-prescribing among young doctors, and does it differ according to gender, type of medication, and type of work? Are there any changes in these patterns during the first postgraduate years?

• Is there an association between help-seeking behaviour for mental disorders and self-prescribing of medication in the postgraduate years?

• Can age, gender, self-prescribing during internship, help-seeking, somatic complaints, subjective health complaints, and mental distress predict self-medication?

## Methods

### Participants

A cohort, consisting of all medical students graduating from all four Norwegian medical schools in 1993–94 (*N *= 631), was sent a postal questionnaire three times: at the end of the internship year (one year after graduation) (T1), at the end of the fourth postgraduate year (T2), and in the 10 th year after graduation (T3).

At T1, 402 responded (64% of the original cohort); their mean age was 29 years (SD 2.8) and 56% were women. At T2, 422 responded (67%); their mean age was 31.4 years (2.4) and 56% were women. At T3, 390 responded (62%); their mean age was 37 years (2.7) and 58% were women. Across the entire study period, 252 (40%), 55% of whom were women, responded at all observational times.

### Use of prescription medication

At T1, the residents were asked "Have you ever treated yourself with a prescription medication?" with the following response alternatives: "No, I have not had the need"; "No, I received it from another physician"; "Yes, on one or two occasions"; "Yes, sometimes"; and "Yes, often". Because medical students in Norway receive a temporary medical licence in their 10 th semester, the word "ever" indicates a lifetime prevalence of self-prescribing of 2.5 years. At T2 and T3, the prevalence of self-prescribing over the previous year was explored using the same response alternatives.

### Prevalence of self-prescribing

At all three times, the participants were asked: "Do you prescribe at present, or have you previously prescribed, some of the following medications for yourself?" with the response alternatives: "Never"; "Yes, previously"; and "Yes, at present". The prevalence of self-medication for the different medications was determined by combining the two final responses. The following medications were asked about: antibiotics, contraceptive pills, migraine medications, prescription analgesics, antihypertensive medications, hypnotics, sedatives, and other psychotropic medications.

### Type of work

At T2, the physicians were asked to report in which specialization they were working, according to a list of 53 medical specializations in Norway. At T3, 10 response alternatives of work situation were given, such as hospital physician with or without leader responsibilities, community physician, specialist in private practice, general practitioner, full-time researcher. Based on these categories, the physicians were divided into the following three main groups of work location: hospital physicians, general practitioners (including specialists in private practice), and others (includes all types of preclinical medicine, laboratory medicine, and full-time research).

### Self-prescribing and mental health problems

At T2 and T3, the participants were asked: "If you had mental health problems during the last year, did you seek or receive help for them?" [13]. The response alternatives were: "Have had no mental health problems of importance"; "Have not sought help, although I have needed it"; "Yes, have consulted a general practitioner"; "Yes, have consulted a psychologist or psychiatrist"; and "Yes, have been admitted to a hospital psychiatric department". Participants who had needed treatment responded with one of the four latter answers, whereas those who had sought help answered with one of the three latter categories.

### Help-seeking

At T3, the participants were asked: "Have you, during the last year, consulted some of the following health care workers with your own problems?" Among the nine response categories was "General practitioner" ("No" or "Yes").

### Somatic complaints

Somatic complaints at T3 were measured by one question: "Do you suffer, or have you suffered, during the past year from ..." with the following eight items as answers: "menstrual pain", "impaired vision", "asthma, lung disease or respiratory disease", "allergy or skin problems", "cardiovascular disease or hypertension", "diabetes", "disease in kidneys or urinary tract", and "gynaecological problems or gynaecological disease". The items were scored on a five-point scale ranging from "not at all" (0) to "very much" (4). An index of the eight items was calculated by summing the responses.

### Subjective health complaints

The subjective self-evaluation of health was assessed at T3 by a 10-item version of the Subjective Health Complaint (SHC) questionnaire [29,30]. This questionnaire consists of questions examining the occurrence, intensity, and duration of musculoskeletal pain, migraine or headache, and digestive problems for the previous 30 days [31]. The items are scored on a four-point scale ranging from "no complaints" (0) to "serious complaints" (3). Seven of the 10 items are related to musculoskeletal symptoms. The sum of the scores of all items was used to indicate the level of subjective health complaints.

### Mental distress

Mental distress was measured at T3 using the SCL-5, a five-item version of the Symptom Check List-25, which captures anxiety and depressive symptoms [32-34]. The SCL-5 includes a question about how much one has been bothered by the following distress items during the last 14 days: "Feeling fearful"; "Nervousness or shaking inside"; "Feeling hopeless about the future"; "Feeling blue"; and "Worrying too much about things". Each item was measured on a five-point scale, from "not at all" (0) to "very much" (4). The sum of the scores of all items was used to indicate the level of mental distress.

### Ethics

To ensure confidentiality, the questionnaire was answered so that the identity of respondents remained anonymous to the researchers, and the name and address codes were kept in Statistics Norway. The study was conducted in collaboration with the Norwegian Medical Association and with the approval of the Norwegian Data Inspectorate.

### Statistical analyses

Chi-square (5% level of significance) was used to test for differences between categorical variables. Logistic regressions were used to test the associations between the predictor variables and the dichotomous outcome measure. Odds ratios (ORs) are presented with 95% confidence intervals in parentheses.

## Results

As shown in Table 1, most of the physicians reported that they had self-prescribed medication. At the end of internship (T1), 69% reported lifetime self-prescribing, which comprised the preceding 2.5 years. The figures were somewhat lower at T2 and T3 (about 54%), because only the previous year's prevalence was asked at this time. At all three times, 10% or fewer had obtained all necessary medication from another physician. Most (74%–81%) physicians who had not self-prescribed (non-prescribers) had not needed drugs to treat themselves. Of the physicians using prescription medication, 90% (T1), 86% (T2), and 84% (T3) had self-prescribed the drugs.

**Table 1:** Prevalence of self-reported prescription behaviour among young physicians

	Have not used prescription medication	Have got all prescription medication from another physician	Have self-prescribed on one or two occasions	Have self-prescribed sometimes	Have self-prescribed often
First postgraduate year (T1) # *n *= 397	90 (22.7%)	31 (7.8%)	215 (54.2%)	57 (14.4%)	4 (1.0%)
Fourth postgraduate year (T2) ## *n *= 422	158 (37.4%)	38 (9.0%)	177 (41.9%)	37 (8.8%)	12 (2.8%)
Ninth postgraduate year (T3) ## *n *= 387	136 (35.1%)	40 (10.3%)	162 (41.9%)	43 (11.1%)	6 (1.6%)

# At T1, lifetime prevalence implies the last 2.5 years, since the students are allowed to prescribe medicaments from the 10 th semester as a part of their temporary medical license

## last year prevalence

The prevalence of self-prescribing did not differ significantly between men and women, or by location of work at any of the three times. At T2 and T3 (previous year), 52% and 54% of the women, and 57% and 56% of the men, reported that they had self-prescribed. Among those who had taken prescription medication during the past year, the prevalence of self-prescribing did not differ between men and women. At T2 and T3, 85% and 83% of the women, and 85% and 88% of the men, had self-prescribed during the previous year.

Table 2 shows that the most frequently self-prescribed medications at the three times were antibiotics, contraceptives, analgesics, and hypnotics. Sedatives were self-prescribed by 3.1% (T3) of the physicians, and other psychotropic medications by 0.8%. Among women, 39% (T1), 43% (T2), and 40% (T3) had self-prescribed contraceptives.

**Table 2:** Prevalence of self-prescription at the first (T1), the fourth (T2) and the ninth (T3) postgraduate years

	T1 *lifetime*	T2 *last year*	T3 *last year*
	*n *(%)	*n *(%)	*n *(%)

Antibiotics	210 (53.0)	299 (71.0)	312 (80.6)
Contraceptive pills	88 (23.0)	102 (24.8)	90 (23.9)
Prescription analgesics	73 (18.5)	74 (17.7)	81 (20.9)
Hypnotics	26 (6.6)	39 (9.3)	48 (12.4)
Sedatives	4 (1.0)	11 (2.6)	12 (3.1)
Migraine medications	12 (3.1)	11 (2.6)	5 (1.3)
Other psychotropic medications	2 (0.5)	7 (1.7)	3 (0.8)
Antihypertensive medications	1 (0.3)	3 (0.7)	1 (0.3)

The prevalence of self-prescribing did not differ significantly between hospital physicians and general practitioners at T2 or T3. At T2, 54% of the hospital physicians and 56% of the general practitioners reported that they had self-prescribed medication during the previous year. At T3, 56% of both groups had self-prescribed.

Those who had needed treatment for mental problems had self-prescribed hypnotics and sedatives to a greater extent than the others, both at T2 (χ^2 ^= 13.0, *P *< 0.001) and at T3 (χ^2 ^= 11.8, *P *< 0.001). However, among those with mental problems needing treatment, those who had sought help did not differ from the others at any time point.

Table 3 shows the adjusted predictors for self-prescribing behaviour. At T3, being male, having self-prescribed during internship (T1), and having contemporary subjective health complaints, somatic complaints, mental distress, and not having consulted a general practitioner during the preceding year were significant predictors of self-prescribing sometimes or often. We performed multiple regression analyses separately for men and women. None of the variables were significant predictors in women, whereas all the variables in the model, except for mental distress and age, were significant predictors in men.

**Table 3:** Predictors of self-prescribing nine years after graduation (T3) among 252 Norwegian physicians

	Crude Odds Ratio (95% CI)	Adjusted Odds Ratio (95% CI)
Age	1.07 (0.97–1.18)	1.09 (0.94–1.25)
Gender (1 = male)	1.6 (0.9–3.0)	3.3 (1.3–8.5) *
Self-prescription at T1	3.1 (1.4–6.8) **	2.7 (1.01–7.4) *
Somatic complaints at T3	1.4 (1.2–1.7) ***	1.5 (1.1–1.9) **
Mental distress at T3	1.2 (1.07–1.24) ***	1.13 (1.01–1.25) *
Subjective health complaints at T3	1.2 (1.1–1.3) ***	1.2 (1.08–1.4) **
Sought general practitioner (last year) at T3	0.9 (0.5–1.8)	0.2 (0.1–0.7) *

* *P *< 0.05.

** *P *< 0.01.

*** *P *< 0.001.

## Discussion

There are several important findings from our study. Most of these young physicians had self-prescribed at least once during the previous year, and this behaviour started early in their careers and persisted at a relatively high level throughout the follow-up period. The most frequently self-prescribed medications were antibiotics, contraceptives, analgesics and hypnotics. Those who had mental health problems needing treatment were more likely to self-prescribe hypnotics than those without perceived mental health problems. Being male, having self-prescribed during internship, somatic complaints, mental distress, subjective health complaints, and lack of seeking help from a general practitioner were significant predictors of self-prescribing in the ninth postgraduate year.

The relatively high level of self-prescribing in our study concurs with the findings in other countries [4,28]. However, direct comparisons with other studies are difficult because of different questionnaires and time frames [1-6]. Previous studies have shown that physicians often self-prescribe medications, most practise self-treatment when they are ill, many have problems accepting their own illness, and many tend to avoid taking sick leave during an illness for which they would have sick-listed their patients [35]. However, this illness behaviour may be changing. For example, the sick-leave rate among physicians is rising [36,37]. This may indicate that their health is getting worse or that they have started to take better care of their own health.

We found no differences in self-prescribing behaviour between hospital physicians and general practitioners. One possible reason is that this cohort is young and that differences may be revealed later in their careers. Alternatively, this trend might relate to a similar pattern of self-prescribing behaviour among general practitioners and hospital physicians. We also found no difference in the self-prescribing behaviour between men and women, which agrees with previous data showing only small gender differences among physicians. However, being male was a significant predictor of self-prescribing in the ninth postgraduate year.

Physicians start self-prescribing early in their careers and at a relatively high level, which seems to be stable over the first postgraduate years. These findings are novel and have not been reported previously in longitudinal studies, although they are expected from the trends in previous cross-sectional studies [4,5,38,39]. Interestingly, in a recent study of medical students in London, most agreed that it is appropriate for doctors to self-investigate and self-refer, but fewer approved of doctors self-prescribing [39]. Presenting information about the dangers of self-prescribing in medical school and having medical students reflect on this topic might help reduce the prevalence of self-prescribing behaviour among physicians. Altering the climate of stoicism at an early stage in the medical career [3,40], and incorporating personal and professional development as part of the curriculum, might provide an opportunity to address issues of self-care [41].

Participants needing treatment for mental problems self-prescribed hypnotics and sedatives to a greater extent than the others. Among those with mental problems needing treatment, those who had sought help did not differ from the others at any time. However, the number of individuals was low. Physicians with mental health problems may be a target group for intervention. The most frequently self-prescribed medications in our sample were consistent to some extent with previous findings [4,5,24]. Few respondents reported that they self-prescribed sedatives or other psychotropic medications. However, those who do may be of concern.

One aim of our study was to determine whether subjective health complaints, mental distress, and somatic diseases predicted self-prescribing behaviour; the multivariate model showed that each of these was an independent predictor for self-prescribing. Interestingly, self-prescribing in internship was also an independent and significant predictor for self-prescribing in the ninth postgraduate year. This shows that the habit of self-medication starts early and supports the view that medical school is a relevant time to introduce students to the concepts of ethical thinking and reflection on this topic. Seeking help from a general practitioner was associated with less self-prescribing when we controlled for other predictors. This underpins the notion that more adequate help-seeking behaviour may reduce the level of self-prescribing.

Most papers view self-prescribing among physicians as a problem. Unfortunately, we do not have data to determine whether the self-prescribed medicaments were re-prescriptions of medicaments initiated by another physician, or if the self-prescription was totally self-initiated. It may be appropriate for physicians to self-prescribe under some circumstances [4,42], for example, to renew a prescription for long-term medication initiated by another doctor (e.g. contraceptives). It may also be appropriate for physicians to self-prescribe for minor illnesses, such as antibiotics for trivial infections. Such prescribing may also be practical and time saving in some parts of Norway because of the long distances between physicians. However, we did not identify any differences in self-prescribing behaviour between general practitioners and others.

### Strengths and limitations

To our knowledge, this is the first longitudinal study of self-prescribing by physicians that followed the course and development of self-medication over time. One strength is the study's prospective design, because it allowed us to identify risk factors for self-prescribing behaviour. The validity of the data is reinforced by the study design, which used a nationwide, representative sample of an occupational group followed for nine years. Another strength is the inclusion of several previously validated instruments in the comprehensive questionnaire. The main findings may be generalized to other countries, because medical doctors belong to a relatively homogenous cross-national occupational group. A limitation of the study is that the response rates were not high in the longitudinal sample, and the results relied solely on self-report because no central prescription register was available. Some level of report bias may have occurred, because physicians may view self-prescribing as socially undesirable, at least as related to psychotropic medication.

## Conclusion

The prevalence of self-prescribing was relatively high in this cohort of young physicians. Most self-prescribed medications are not addictive or prescribed for chronic or mental disorders. However, a substantial proportion of the respondents self-prescribed hypnotics. Moreover, those who self-prescribed early in their career were more prone to self-prescribe later on. These findings suggest that the issue of self-prescribing probably should be addressed in medical school.

## Competing interests

The author(s) declare that they have no competing interests.

## Authors' contributions

EH was involved in designing the study, analysing the data and writing the paper. GS was involved in analysing the data and writing the paper. RT was involved in designing the study, analysing the data and writing the paper. NTG was involved in initiating and designing the study, was responsible for the collection of data, and entered the data into the computer. PV and ØE initiated and designed the study and supervised the collection of data, and were involved in writing the paper. EH is the guarantor.

## Pre-publication history

The pre-publication history for this paper can be accessed here:


